# Aspergillus fumigatus keratitis following intracorneal ring segment implantation

**DOI:** 10.1186/1471-2415-12-19

**Published:** 2012-07-06

**Authors:** Wisam A Shihadeh

**Affiliations:** 1 Department of ophthalmology, Faculty of medicine, Jordan University of Science & Technology, Irbid, Jordan

**Keywords:** Fungal, Aspergillus, Keratitis, Intracorneal ring segment

## Abstract

**Background:**

Fungal keratitis has been rarely reported following intracorneal ring segment (ICRS) inmplantation. This paper aims to report a case of fungal keratitis with aspergillus fumigatus following ICRS implantation for correction of keratoconus.

**Methods:**

A retrospective chart review was done. Data including demographics, clinical history and presentation, microbiological analysis as well as clinical management were recorded.

**Results:**

A 34 year old male presented with pain, photophobia, redness and decreased vision in his right eye ten days after ICRS implantation for correction of keratoconus. Slit-lamp examination showed chemosis, ciliary injection, corneal abcess with ill defined edges and hypopyon. Microbiological analysis and culture of the corneal scrapes were positive for aspergillus fumigatus. The patient did not respond to medical treatment and ended up with corneal transplantation.

**Conclusion:**

Although rare, fungal keratitis is a serious vision threatening complication that can complicate intrastromal ring implantation. Prompt and aggressive treatment is essential to prevent irreversible reduction of vision.

## Background

Intracorneal ring segment (ICRS) implantation is one of the current modalities for correction of keratoconus [1]. Microbial keratitis has been reported previously following this procedure [2-5]. Bacterial keratitis was the commonest entity; Staphylococcus and Streptococcus were the predominant organisms isolated [5,6]. Although aspergillus species were described before as a cause of keratitis [7], it was never reported –up to our knowledge- following ICRS implantation. We report a case of aspergillus keratitis following an uncomplicated implantation of an ICRS (CornealRing^TM^, Visiontech, Brazil) to correct keratoconus.

## Case presentation

A 34 year old gentleman was referred to our department with bilateral keratoconus. He had moderate myopia and astigmatism and was intolerant to contact lenses. Sequential ICRS implantation was done in both eyes. Postoperatively, the patient was described antibiotic, steroid and lubricant eye drops. The left eye (first eye done) had an unremarkable postoperative course. Ten days following ICRS implantation in his right eye, the patient developed redness, pain, photophobia and deterioration of vision in a slowly progressive fashion. The patient mentioned he had to refit the contact lens that fell down on the floor. He mentioned also that he washed it with an expired contact lens solution. Unfortunately, we could not retrieve that solution for microbiologic analysis.

**Figure 1:**
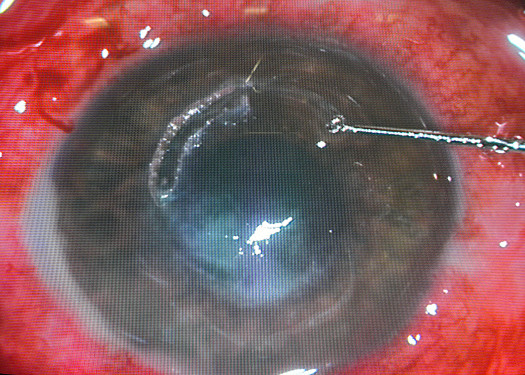
Intracorneal ring segment explanation following aspergillus keratitis.

**Figure 2:**
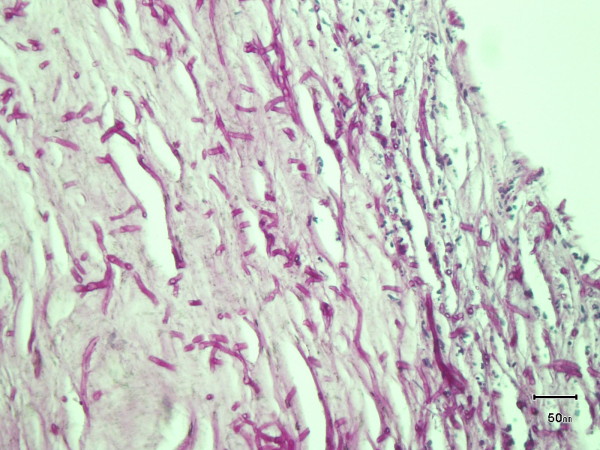
PAS stain showing septate hyphae with acute branching consistent with aspergillus species.

Examination showed a corneal abcess with ill defined edges, hypopyon with severe chemosis and ciliary injection. KOH smear and culture were positive for aspergillus fumigatus. The patient was admitted and treated initially with gatifloxacin eye drops on hourly basis. Systemic oral itraconazole (200 mg/day in two divided doses) and topical amphotericin B 0.15% eye drops (hourly around the clock) were added when the results of the microbiological analysis came out. The ring was explanted as well (Figure 1) few days after presentation. There was no response to all these measures and the cornea started to melt. Collagen cross linking was tried but again without significant clinical response. An uneventful therapeutic penetrating corneal transplantation was done a month after the onset of the infection. Histopathology results of the excised cornea confirmed the diagnosis (Figure 2). Postoperative follow up for 16 weeks showed a clear graft with 0.3 visual acuity. No recurrence of the infection was seen so far.

## Discussion and conclusion

Patients with keratoconus usually have difficulty achieving satisfactory vision with glasses. Also, not uncommonly they are intolerant to contact lenses. The efficacy of ICRS implantation for the correction of keratoconus has been demonstrated [1]. Microbial keratitis is a serious sight threatening condition. Although infection after ICRS implantation is uncommon, it has been described in more than one report [2-5]. The consequences of this infection could serious; Hofling-Lima et al. described in his series two patients that required penetrating keratoplasty [3]. Some of the possible risk factors described included diabetes, trauma, and the use of contact lenses after implantation of ICRS [2]. Any pathogen can potentially cause this sight-threatening complication; microorganisms identified include Staphylococcus aureus, Staphylococcus epidermidis, Streptococcus viridans, Streptococcus pneumoniae, Clostridium perfringens, Pseudomonas species, Nocardia species, Klebsiella species, and Paecilomyces species [2-4]. Fungal keratitis constitutes a challenge for ophthalmologists both in diagnosis and treatment. It is not common to isolate the organism and the diagnosis is usually based on the clinical presentation.

Our case – up to our knowledge- is the first case of aspergillus keratitis following ICRS implantation. Trauma and the use of contact lens that was potentially contaminated are the two risk factors that we could identify in our case. We were lucky to isolate the organism because the clinical picture was not specific. Although fungal keratitis is usually resistant to medical treatment, successful outcome has been reported with the use of topical and intrastromal voriconazole [8]. In addition to early and aggressive medical treatment, ICRS should be explanted. The role of collagen cross linking for the management of infectious keratitis is still being studied. Therapeutic penetrating keratoplasty is the ultimate option should the conservative methods fail to control the infection.

To conclude, fungal keratitis - although rare- is considered a serious vision threatening complication that can develop following ICRS implantation. Prompt and aggressive treatment is essential to prevent irreversible reduction of vision.

## Consent

A written informed consent was obtained from the patient to publish this case report.

## Competing interests

The author declares that they has no competing interests.

## Disclosure

The author has no conflict of interest with the submission.

## Financial support

No financial support was received for this submission.

## Pre-publication history

The pre-publication history for this paper can be accessed here:

http://www.biomedcentral.com/1471-2415/12/19/prepub
